# Proteomic profiling of lysine acetylation and succinylation in *Staphylococcus aureus*

**DOI:** 10.1002/ctm2.1058

**Published:** 2022-09-30

**Authors:** Jingyan Xia, Jinliang Liu, Feng Xu, Hui Zhou

**Affiliations:** ^1^ Department of Oncology Radiation Second Affiliated Hospital, Zhejiang University School of Medicine Hangzhou China; ^2^ Department of Infectious Diseases Second Affiliated Hospital, Zhejiang University School of Medicine Hangzhou China; ^3^ Research Center for Life Science and Human Health Binjiang Institute of Zhejiang University Hangzhou China

Dear Editor,

Although antimicrobial treatment of *Staphylococcus aureus* (*S. aureus*) infection is standard therapy for patients, mortality remains high as for the complications which include myocarditis.^1^ Understanding the regulation of the proteome in the *S. aureus* bacterium may provide new insights in the treatment of this bacterial infection and also new basic knowledge of molecular signalling. Post‐translational modification (PTM) of the lysine residue has significant consequences for functional and regulatory implications, including cytoskeleton complexes.^2^ Specifically, *N*‐lysine acetylation (Kac) and succinylation (Ksucc) prove to act as important PTMs for regulating various biological processes, including amino acid metabolism, protein translation and energy metabolism.^3^, ^4^ In this study, we systematically investigated lysine acetylation and succinylation sites in *S. aureus* using mass spectrometry (Supporting Information) and identified 1778 lysine acetylation sites from 794 proteins and 1651 lysine succinylation sites from 450 proteins. The finding, to our knowledge, is the largest number of acetylated and succinylated proteins to be discovered in *S. aureus*.

Among the 794 lysine acetylated proteins in *S. aureus*, 48.1% and 6.3% have one or over five acetylated sites, respectively. We identified the top three proteins to be most poly‐acetylated, which were oligopeptidase F (17 sites), phosphoglycerate kinase (11 sites) and elongation factor G (10 sites). For 450 lysine succinylated proteins, 33.3% were singly modified and 24.2% were poly‐succinylated. The top three poly‐succinylated proteins were bifunctional autolysin (23 sites), foldase protein PrsA (20 sites) and phosphoglycerate kinase (19 sites) (Figure 1A,B).

**FIGURE 1:**
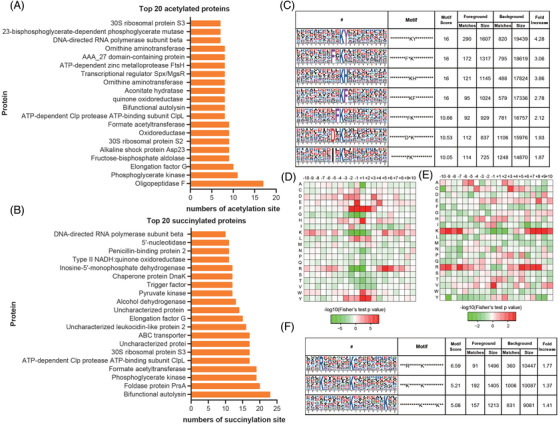
Acetylation and succinylation examination and motif analysis of Kac and Ksucc sites in proteins of *Staphylococcus aureus*: (A) top 20 acetylated proteins, (B) top 20 succinylated proteins, (C) peptide motifs with conserved residues around Kac sites, (D) heat map indicating amino acid residues around Kac sites in identified peptides, (E) heat map indicating amino acid residues around Ksucc sites in identified peptides and (F) peptide motifs with conserved residues around Ksucc sites.

To further characterize Kac and Ksucc sites, we examined the sequence motif around the identified peptides. Highly conserved motifs were found to be matched in the identified acetylated peptides (Figure 1C,F). These highly conserved motifs are consistent with previous reports, including eukaryotes, where Kac‐tyrosine (Y) and Kac‐histidine (H) motifs were reported.^5^, ^6^ A heat map further showed that glycine (G), lysine (K) and arginine (R) were significantly under‐represented in the −2 to +1 positions surrounding the acetylated lysine, whereas phenylalanine (F), Y and K were significantly over‐represented at the −2 to +2, −3 to +2, −10 to −7 and +6 to +10 positions, respectively (Figure 1D). At the positions surrounding the succinylated lysine, the residues K and R were significantly over‐represented at the positions of −10 to −7, +6 to +10 and −10 to −7, +5 to +8, with less presentation at the −1 to +2 and −1 to +1 positions, respectively (Figure 1E). The preferential representation of phenylalanine at the −2 to +2 positions surrounding the acetylated lysine appears to be unique feature for *S. aureus*, considering the variety of acetyltransferases preferences adopted by different bacteria.^7^

We then performed gene ontology (GO) term analysis on the modified proteins. The biological process analysis revealed that acetylated enzymic proteins and succinylated enzymic proteins are enriched with protein related to the metabolism process (40%; 39%) and cellular process (33%; 35%). The molecular function analysis revealed that acetylated and succinylated proteins were highly related to catalytic activity (48%; 42%) and binding (40%; 42%). For cellular component category, most acetylated proteins belonged to the intracellular protein category (59%), followed by the macromolecular complex (14%), membrane (14%), organelle (10%) and extracellular compartments (3%). Similarly, the majority of succinylated proteins were located intracellularly (54%), the macromolecular complex (19%) and organelle compartments (15%) (Figure 2A–F).

**FIGURE 2:**
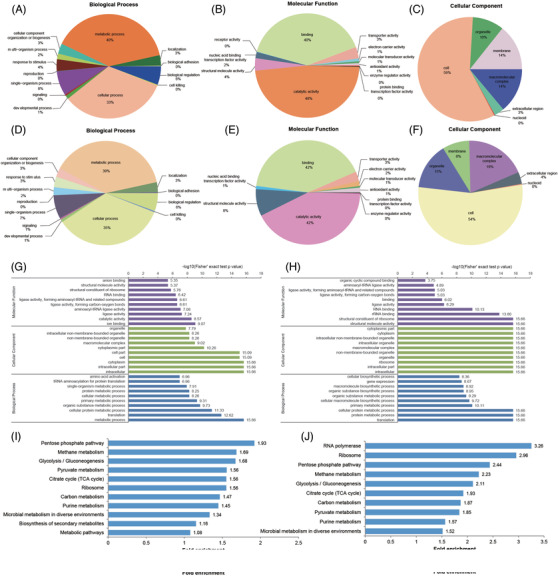
Functional classification and gene ontology (GO) enrichment analysis of proteins with lysine acetylation and succinylation in *Staphylococcus aureus*. GO classification of acetylated proteins based on biological process (A), molecular function (B) and cellular component (C). GO annotation of succinylated proteins based on biological process (D), molecular function (E) and cellular component (F). The acetylated (G) and succinylated (H) proteins were grouped by their GO annotation regarding molecular function (purple bars), cellular component (green bars) and biological process (blue bars). The KEGG pathway enrichment analysis of acetylation (I) and succinylation (J). The top *x*‐axis indicates the values of the folds enrichment as indicated in the left *y*‐axis

Enrichment analyses of GO, protein domain and Kyoto Encyclopedia of Genes and Genomes (KEGG) pathway were then carried out to more clearly define the properties of acetylated proteins and succinylated protein. It was revealed that cellular protein metabolic process and translation were extraordinarily enriched for both acetylation and succinylation. To be functional relevant, the top two acetylated proteins were linked with ion binding and catalytic activity. Moreover, structural molecule activity and structural constituent of ribosome were significantly enriched in succinylated proteins. The analysis on cellular components revealed that intracellular part and cytoplasm were significantly enriched for acetylated proteins. Conversely, the succinylated proteins appeared in diverse cellular compartments. These results indicated that both acetylated and succinylated proteins were linked with a broad cellular component possessing a wide spectrum of molecular functions and biological activities (Figure 2G,H).

The protein domain analyses of acetylated proteins revealed that nucleic acid‐binding, class I and II aminoacyl‐tRNA synthetase appeared to be more acetylated (Figure S1A). Significantly, succinylated proteins included class I aminoacyl‐tRNA synthetase and nucleic acid‐binding (Figure S1B). Besides, thioredoxin‐like fold, translation protein SH3‐like domain and pyridine nucleotide‐disulphide oxidoreductase, the nicotinamide adenine dinucleotide (NAD)‐binding domain were also prominent.

The KEGG pathway analysis revealed that the pentose phosphate pathway, methane metabolism and glycolysis/gluconeogenesis were enriched in acetylated proteins. Moreover, many enzymes related to metabolic pathways were acetylated, such as tricarboxylic acid (TCA) cycle and pyruvate metabolism. RNA polymerase, ribosome and pentose phosphate pathway were among the top three pathways in succinylated enzymes (Figure 2I,J and Figures S2–S5). In combination, the results revealed that cellular metabolism and molecular binding are the most important physiological activity of lysine acetylated and succinylated proteins in *S. aureus*, in‐line with the observation in other bacterial species.^5^, ^8^

The protein–protein interaction networks of all known acetylated proteins and succinylated proteins have demonstrated that there were 607 acetylated proteins and 252 succinylated proteins as nodes, with 2828 and 2009 direct physical interactions discovered in a wide range of biological processes and protein–protein interaction networks, respectively (Figures S6A and S7A). The top five significantly enriched function clusters of acetylated proteins included the ribosome, glycolysis/gluconeogenesis, purine metabolism, pyruvate metabolism and aminoacyl‐tRNA biosynthesis (Figure S6B–F). The top three significantly enriched clusters of succinylated proteins were ribosome, pentose phosphate pathway and aminoacyl‐tRNA biosynthesis (Figure S7B–D). The overlap analysis between acetylated and succinylation peptides showed 382 proteins with overlapping modifications, specifically, 84.9% succinylated and 48.1% acetylated (Figure 3). The top two density subnetworks included ribosome and carbon metabolism. The data, along with GO enrichment and KEGG pathway analysis, indicated that these complexes’ physiological interactions may promote the coordinated biological process in *S. aureus*. Our result presented the first extensively interaction network of the acetylated and succinylated proteins in *S. aureus*.^9^

**FIGURE 3:**
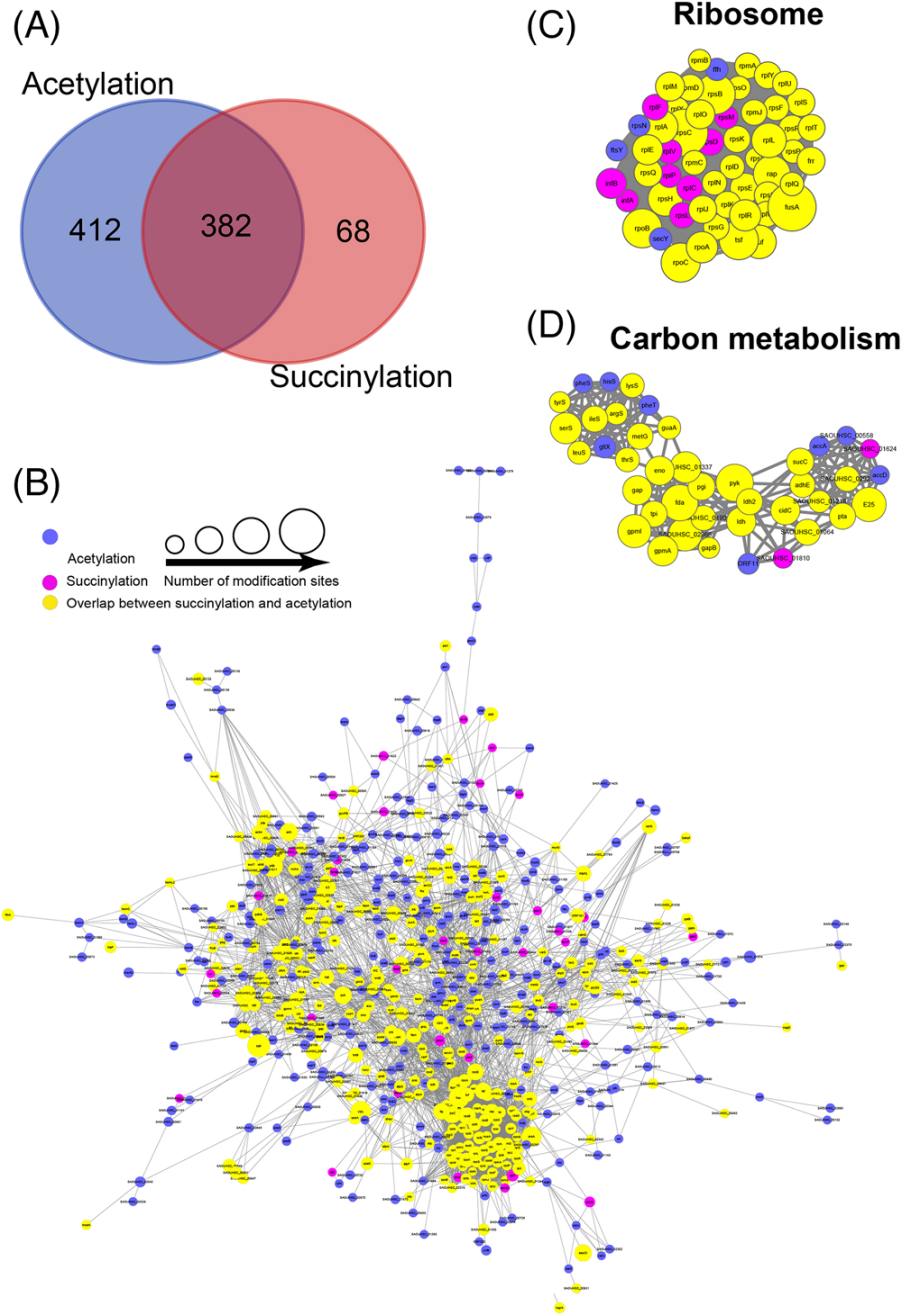
Overlapping between lysine acetylation and succinylation of proteins in *Staphylococcus aureus*: (A) the number of proteins with acetylation, succinylation or both, (B) the overlap diagram between lysine acetylation and succinylation. The most overlapped function clusters were related with ribosome (C) and carbon metabolism (D). Size of the node indicates the number of modified site in each protein, and the node colour indicates different modified sites, including those with acetylation (blue), succinylation (pink), overlap between acetylation and succinylation (yellow)

A previous study has found that succinylation regulates autolysis and β‐lactam susceptibility in methicillin‐resistant *S. aureus*, suggesting that the modulation of succinylation may affect antimicrobial susceptibility.^10^ Our study also found succinylated protein and penicillin‐binding protein 2, which may influence the antibiotic binding (Figure 1B). Thus, the drugs that modulate acetylation or succinylation will affect a wide range of functions in *S. aureus*, including antibiotic susceptibility. In conclusion, our study reported the largest global investigation of lysine acetylome and succinylome in *S. aureus*. The results pave an avenue for in‐depth exploration of the functions of Kac and Ksucc in the growth, development and pathogenicity of *S. aureus*, which may help the development of new treatment of bacterial infection.

## CONFLICT OF INTEREST

The authors declare no conflicts of interest.

## Supporting information

Supporting Information

## Data Availability

All data that support the findings in this study are available from the corresponding author upon reasonable request.
