# Supplementary material for: Proteomic profiling of lysine acetylation and succinylation in Staphylococcus aureus
Source: Clin Transl Med. 2022 Sep 30;12(10):e1058. doi: 10.1002/ctm2.1058 (PMC9523452; doi:10.1002/ctm2.1058)
Supplement: Supplementary file 1 — Supporting Information [file CTM2-12-e1058-s001.docx]

**Supplymentary**

**Supplymentary 1：**

**MATERIALS AND METHODS**

Protein extraction

As previous research has shown, *Staphylococcus aureus*（Newman）was grown to log phase in TSB broth (TSB, Becton Dickinson, USA) under aerobic conditions at 37°C. Cells were harvested and ground with liquid nitrogen into cell powder and then transferred to a centrifuge tube. Four volumes of lysis buffer (8 M urea, 1% protease inhibitor cocktail, 3 μM TSA and 50 mM NAM) was added to the cell powder, followed by sonication three times on ice using a high intensity ultrasonic processor (Scientz). The remaining debris was removed by centrifugation at 12,000 g at 4 °C for 10 min. Finally, the supernatant was collected and the protein concentration was determined with BCA kit according to the manufacturer’s instructions^[1,2].^

PTM enrichment

The PTMs was enriched based on pan-antibody according to previous methods^[1,3,4]^. For digestion, the protein solution was reduced with 5 mM dithiothreitol for 1 h at 37 °C and alkylated with 15 mM iodoacetamide for 45 min at room temperature in darkness. The protein sample was then diluted by adding 200 mM TEAB to urea concentration less than 2 M. Finally, trypsin was added at 1:50 trypsin-to-protein mass ratio for the first digestion overnight at 37 ℃ and 1:100 trypsin-to-protein mass ratio for a second 4 h-digestion at 37 ℃. The peptides were then desalted by C18 SPE column and vacuum drying. The dried peptides were redissolved in NETN buffer and mixed with pan anti-Kac antibody (PTM-101, PTM Biolabs) or pan anti-Kcuss antibody (PTM-402, PTM Biolabs) agarose beads that had been pre-washed with NETN buffer and subsequently incubated overnight at 4°C with gentle shaking. The beads were then washed three times with NETN buffer and twice with ice-cold ddH_2_O to remove unbound peptides. The beads were eluted by adding 0.1% trifluoroacetic acid (TFA). The eluent was desalted with C18 ZipTips (Millipore) and dried by vacuum concentration^[1,3,4]^.

### LC-MS/MS analysis

### Enrichment of lysine acetylated and succinylated peptides was analyzed using liquid chromatography tandem mass spectrometry (LC-MS/MS) according to the previous methods with some modifications

### ^[2,4]^. Briefly, Kac and Ksucc peptides were separated on a UPLC system using a reversed-phase analytical column (Acclaim PepMap RSLC C18 column, Thermo Scientific). Tryptic peptides were gradient eluted at a flow rate of 250 nl/min. MS/MS with Q Exactive HFX (Thermo Fisher Scientific) connected online to LC was used to detect peptides. In orbitrap, ion fragments were discovered.

QC validation of MS Data

The MS data validation was shown in Figure S1. Firstly, we checked the mass error of all the identified peptides. The distribution of mass error is less than 2 ppm which means the mass accuracy of the MS data fit the requirement for both acetylation and succinylation (Figure 1A and Figure 1C). Secondly, the length of most peptides distributed between 8 and 17 for acetylation (Figure 1B) and between 8 and 20 for succinylation (Figure 1D), which agree with the property of tryptic peptides. That means the sample preparation satisfies the standard both for acetylation and succinylation.


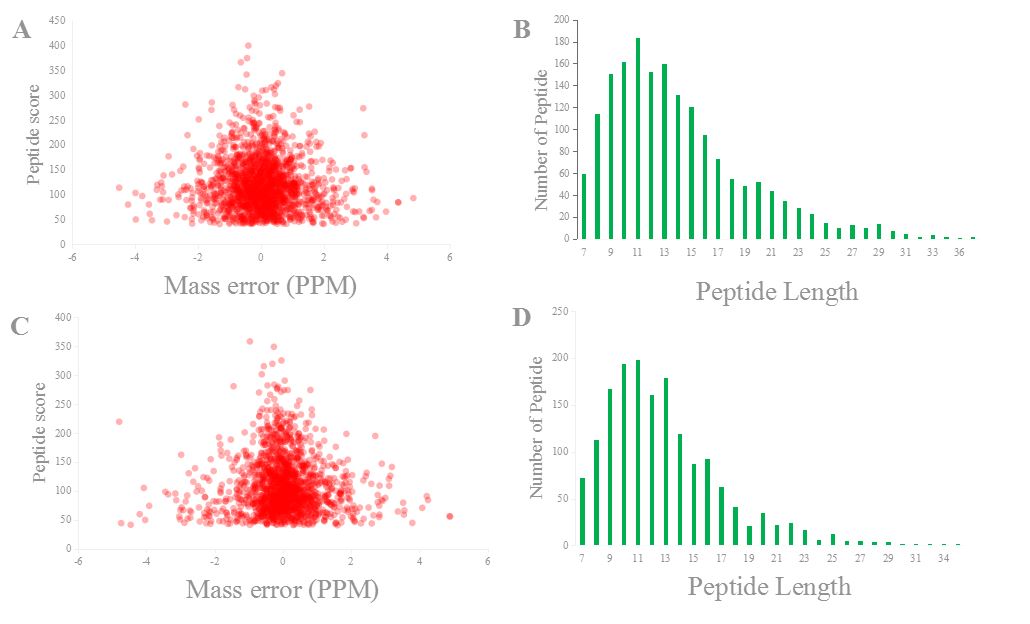


Figure 1. QC validation of MS data. Mass error distribution of all acetylated and succinylated peptides(1A &1C) ; length distribution for all acetylated and succinylated peptide (1B & 1D).

Database Search

The raw data was analyzed using the Mascot engine (Matrix Science, London, UK; version 2.2) incorporated in Proteome Discoverer 1.4 (Thermo Fisher Scientific). Protein sequences from *S.aureus* were chosen as a reference from the UniProt database (http://www.ebi.ac.uk/GOA/). For protein identification, appropriate mass errors of fragment ions and precursor were set.

Bioinformatics Analysis

BLAST2GO was used to extract gene ontology (GO) concepts, and functional annotations were added to the target proteins. Biological process, cellular component, and molecular function were the three categories for modified proteins. STRING (version 10.0) was used to clearly display differentially expressed proteins in the protein-protein interaction network, and the Kyoto Encyclopedia of Genes and Genomes (KEGG) automated annotation system was used to identify metabolic pathways and functional categories. PSORTb v3.0.2 was used to estimate protein subcellular localization distribution.

References：

1. Tu H, Xu F, Cheng Y, et al. Proteomic profiling of the endogenous peptides of MRSA and MSSA. *PeerJ.* 2021; 9:e12508.

2. Gaviard C, Broutin I, Cosette P, et al. Lysine Succinylation and Acetylation in Pseudomonas aeruginosa. *J Proteome Res.* 2018; 17(7):2449-2459.

3. Zhang N, Yang Z, Liang W, et al. Global Proteomic Analysis of Lysine Crotonylation in the Plant Pathogen Botrytis cinerea. *Front Microbiol.* 2020; 11:564350.

4. Xu Y, Li X, Liang W, et al. Proteome-Wide Analysis of Lysine 2-Hydroxyisobutyrylation in the Phytopathogenic Fungus Botrytis cinerea. *Front Microbiol.* 2020; 11:585614.


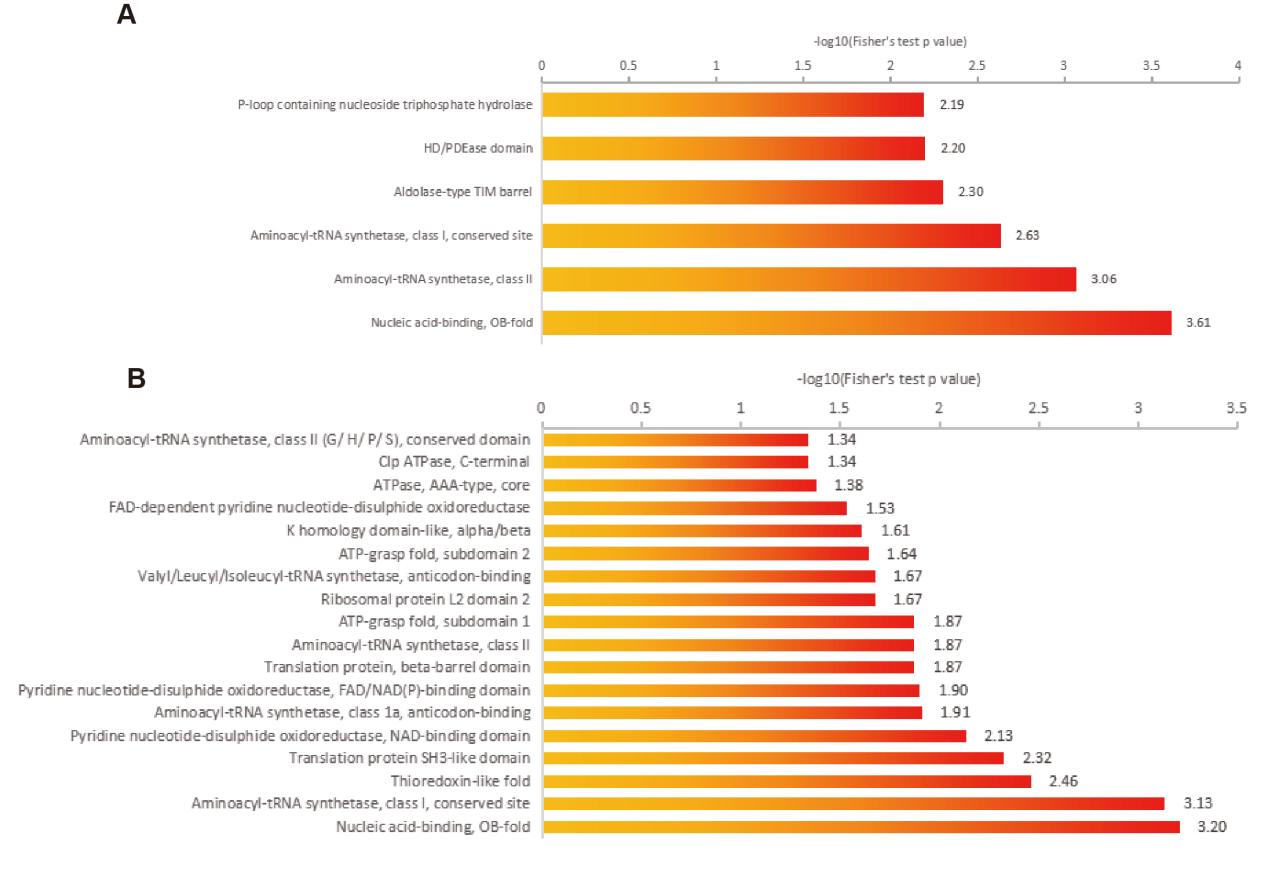


Figure S1. Protein domain enrichment analysis of Acetylation (A) and Succinylation (B). The top x-coordinate indicates value of -log10 (Fisher’s test *p*-value) of the indicating terms in left y-axis.


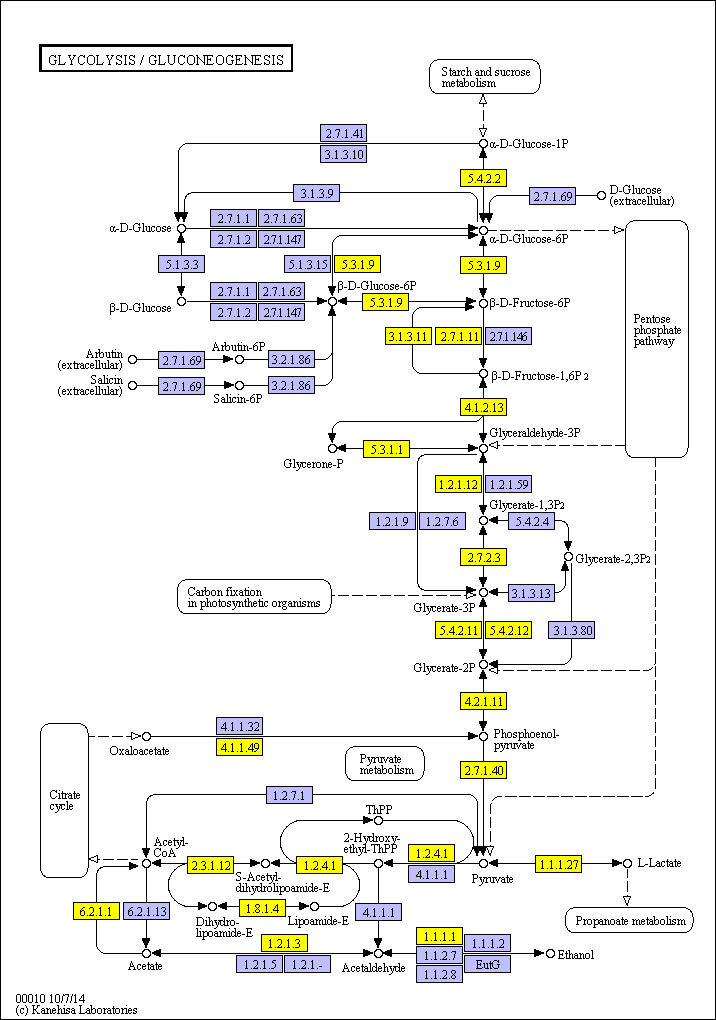


Figure S2. KEGG pathway enrichment of glycolysis/gluconeogenesis of Acetylation.


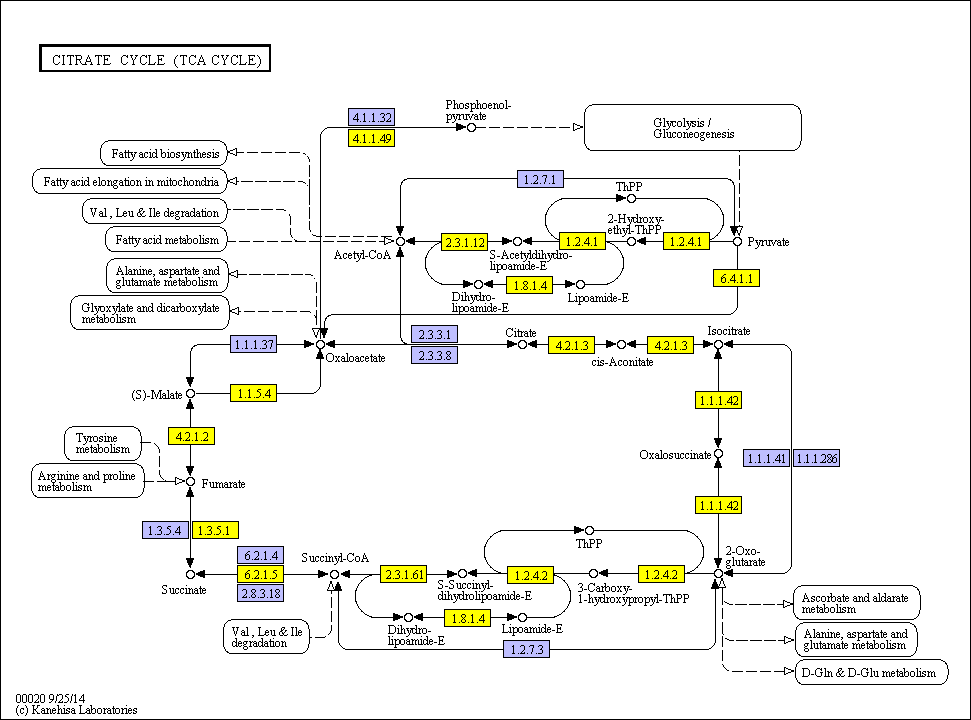


Figure S3. KEGG pathway enrichment of TCA cycle of Acetylation.


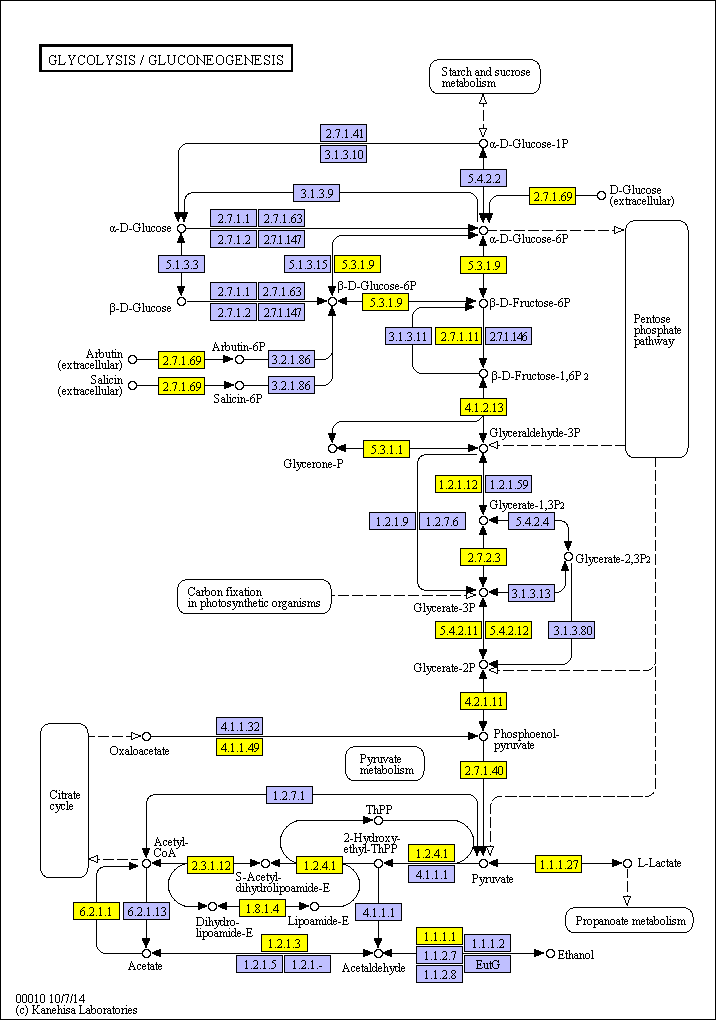


Figure S4. KEGG pathway enrichment of glycolysis/gluconeogenesis of Succinylation.


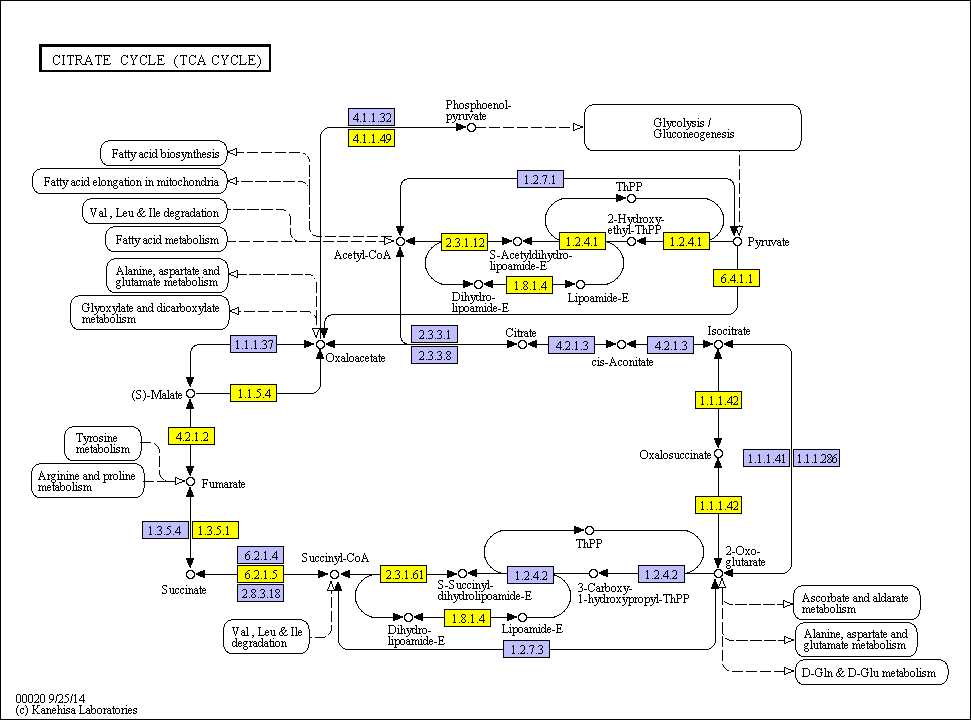


Figure S5. KEGG pathway enrichment of TCA cycle of Succinylation.


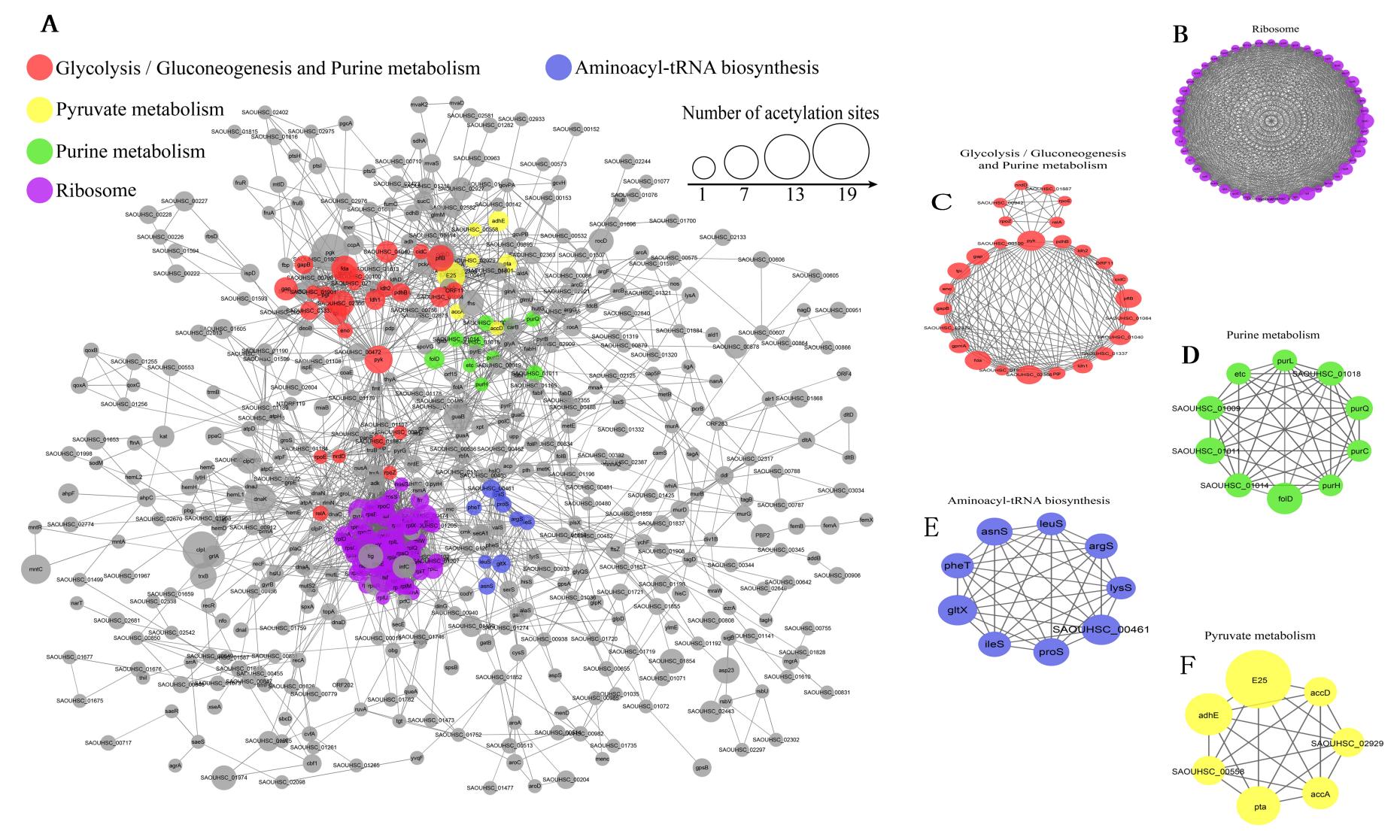


Figure S6. Protein–protein interaction networks of identified acetylated proteins in *S.aureus.* (A). Size of the node indicates number of Kac sites in each protein and node color indicates different enriched function clusters. The top five significantly enriched function clusters with highly interconnected were associated with ribosome (B), glycolysis/gluconeogenesisand purine metabolism (C), purine metabolism (D), pyruvate metabolism (E) and aminoacyl-tRNA biosynthesis (F).


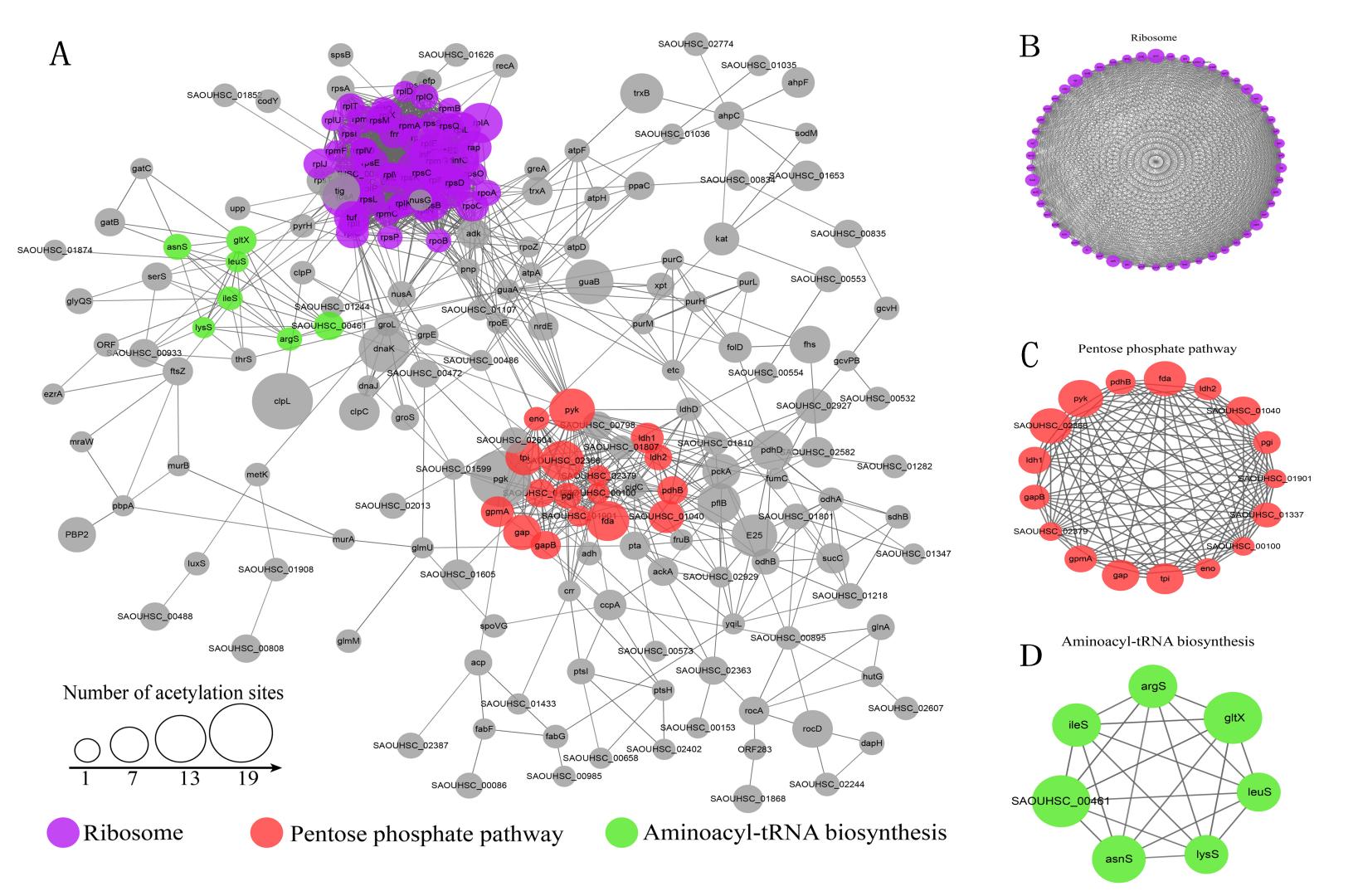


Figure S7. Protein–protein interaction networks of identified *s*uccinylated proteins in *S.aureus.* (A). The top three significantly enriched function clusters with highly interconnected were associated with ribosome (B), pentose phosphate pathway (C) and aminoacyl-tRNA biosynthesis (D). Size of the node indicates number of Ksucc sites in each protein and node color indicates different enriched function clusters.
